# Therapeutic Decision-Making and Outcomes in Elderly Patients With Severe Symptomatic Aortic Stenosis: Prognostic Implications of Elderly Patients' Initial Decisions

**DOI:** 10.3389/fcvm.2021.696763

**Published:** 2021-07-26

**Authors:** Qinghao Zhao, Haiyan Xu, Qingrong Liu, Yunqing Ye, Bin Zhang, Zhe Li, Runlin Gao, Yongjian Wu

**Affiliations:** Department of Cardiology, Fuwai Hospital, National Center for Cardiovascular Diseases, Chinese Academy of Medical Sciences and Peking Union Medical College, Beijing, China

**Keywords:** aortic stenosis, elderly, therapeutic decision making, outcomes, aortic valve replacement

## Abstract

**Background:** Despite clear indications for intervention, therapeutic decision-making for elderly patients with severe symptomatic aortic stenosis (AS) remains a complex issue due to the wide variation in individual risk profiles and the involvement of patients' subjective preferences. We aimed to investigate the reasons leading to the decisions against intervention and the consequences thereof on survival.

**Methods:** Data were derived from the China Elderly Valve Disease (China-DVD) Cohort Study on patients aged ≥60-year-old with severe symptomatic AS consecutively enrolled between September to December 2016. Patients were analyzed according to the initial therapeutic decisions made by consensus between patients and physicians at the time of the index evaluation: intervention group (patients who were evaluated as suitable for intervention and accepted the treatment proposal); patient-refusal group (patients who were evaluated as suitable for intervention but refused due to subjective preferences); physician-deny group (patients who were denied intervention by physicians after evaluation). The least absolute shrinkage and selection operator (LASSO)-penalized logistic regression model was used to identify the factors associated with physicians' decisions against intervention. Twelve-month survival was analyzed using Cox proportional hazards models, with multivariate adjustment using inverse probability weighting (IPW).

**Results:** Among the enrolled 456 elderly patients with severe symptomatic AS, 52 (11.4%) patients refused intervention and 49 (10.7%) patients were denied intervention by their physicians. LASSO-penalized logistic regression model identified that reduced left ventricular ejection fraction and increased EuroSCORE-II were strongly associated with physicians' decisions against intervention. At 12-month follow-up, only 8 (15.4%) patients who initially refused the intervention proposal underwent the subsequent intervention, with an average delay of 195 days. Patients' initial decisions against intervention were significantly associated with 12-month mortality, even after IPW adjustment (Hazard ratio: 2.61; 95% confidence interval: 1.09–6.20; *P* = 0.031).

**Conclusions:** The decision against intervention was taken in about one-fifth of elderly patients with symptomatic severe AS, half of which were due to patients' subjective preferences. Surgical risk remains the primary concern for physicians when making therapeutic decisions. Elderly patients' initial decisions against intervention have a profound impact on subsequent intervention rates and prognosis, and therefore should be treated as a “risk factor” at the subjective level.

**Clinical Trial Registration:** clinicaltrials.gov/ct2/show/NCT02865798, China elDerly Valve Disease (China-DVD) cohort study (NCT02865798).

## Introduction

Aortic stenosis (AS) is a common disease in the elderly, and its prevalence continues to rise as the population ages (1, 2). There is a general consensus that aortic valve replacement (AVR) should be advised in patients with severe symptomatic AS (3, 4). However, therapeutic decision-making remains a complex issue for elderly patients with severe AS due to the wide variation in individual comorbidities and life expectancy (3, 5). Furthermore, patients' subjective preferences also involve and complicate the decision-making process.

Previous studies reported that the decision against intervention was taken by about one-third of elderly patients with severe symptomatic AS, mainly attributed to advanced age and excessive surgical risk (6–8). However, these studies did not distinguish whether the decisions against intervention were made by patients or by their attending practitioners. This may introduce bias into the analysis of therapeutic decision-making, as patients who refused intervention due to subjective preferences were initially evaluated as suitable for intervention, and thus their risk profiles and outcomes were supposed to vary greatly from those of patients who were denied intervention by physicians after evaluation. Moreover, in recent years, the development of the transcatheter aortic valve replacement (TAVR) technique has driven a major paradigm shift in the management of AS, especially enabling AVR to be performed in elderly patients with high or prohibitive surgical risk (3, 4). However, there are little data available to evaluate therapeutic decision-making and outcomes of AS in current clinical practice.

To explore contemporary therapeutic decision-making of elderly patients with severe symptomatic AS and the reasons leading to the decisions against intervention along with the consequences thereof on survival, we conducted the present study using data from the China elDerly Valve Disease (China-DVD) study, a nationwide prospective cohort study enrolling consecutive elderly patients with valvular heart diseases (VHD). An important feature of this study is the detailed documentation of the initial therapeutic decisions at the time of the index evaluation, thereby making it possible to investigate the incidence, clinical correlates, and prognostic impact of the decisions against intervention made by patients and by physicians, respectively.

## Method

### Overview of the China-DVD Study and Study Population

The China-DVD study (NCT02865798) is a nationwide, multicenter prospective cohort study for elderly patients (≥60-year-old) with VHD. The study was conducted from September to December 2016 at 69 large academic hospitals with both cardiology and cardiac surgery departments from 28 provinces and municipalities throughout mainland China, ensuring broad geographic coverage and representation of contemporary status (Supplementary Table 1 and Supplementary Figure 1). The participating sites were instructed to consecutively enroll inpatients with moderate or severe native VHD as defined by echocardiography using an integrative approach according to the 2014 ACC/AHA guidelines (9). Data collection, management, and quality control are detailed in the Supplementary Methods. This project was approved by the Institutional Review Board in each center. Written informed consent was obtained from all eligible participants.

**Figure 1:**
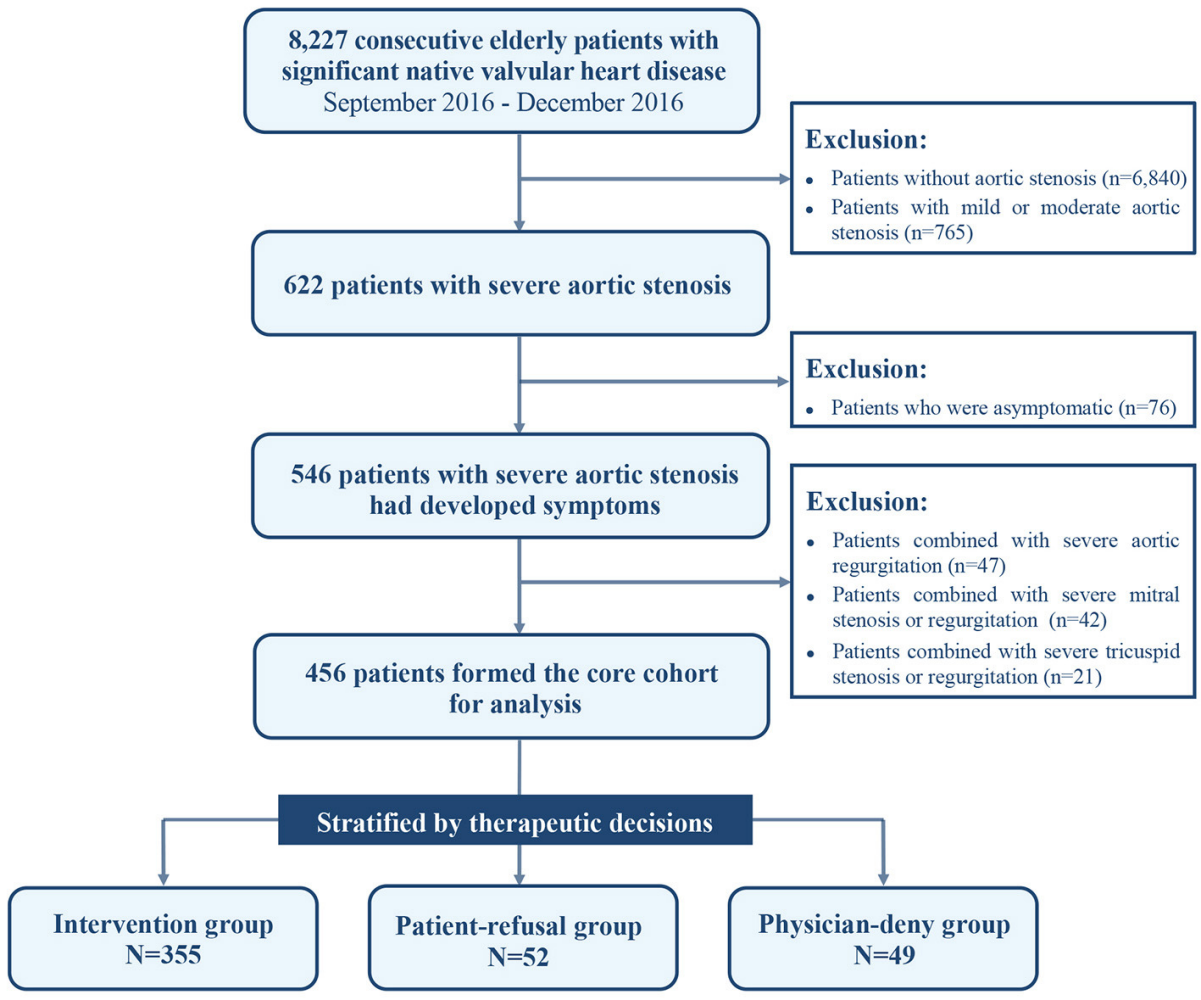
Study cohort flow diagram.

In total, 8,227 patients aged ≥60-year-old with significant native VHD were enrolled in the China-DVD cohort. Severe AS was encountered in 622 patients, as defined by a valve area ≤ 1.0 cm^2^ or a maximal jet velocity ≥4.0 m/s or pressure gradient ≥40 mmHg. Of them, 546 patients had developed the following symptoms either singly or in combination: angina pectoris, syncope, or heart failure with New York Heart Association (NYHA) class II–IV. In this study, we included these elderly patients with severe symptomatic AS and excluded those with associated severe valve disease, including severe aortic regurgitation, and severe mitral or tricuspid valve disease, thus using 456 patients as the core cohort for analysis (Figure 1).

### Clinical Characteristics and Echocardiographic Assessment

Patient characteristics related to demographics, risk factors, comorbidities, symptoms, and investigations were collected. Comorbidities were evaluated individually and combined using the Charlson Comorbidity Index (CCI), a global and validated scoring system that enables comorbidities to be weighted according to prognostic impact (10). The surgical risk was evaluated using the European System for Cardiac Operative Risk Evaluation (EuroSCORE-II) (11), which was calculated as if all patients would have undergone AVR regardless of the actual decisions, thereby allowing the overall surgical risk to be assessed. All patients underwent comprehensive transthoracic echocardiographic evaluation using standard ultrasound systems, including M-mode and 2-dimensional echocardiography as well as Doppler examinations. Dimensions of the left ventricles (LV) and left atrium (LA) were measured as recommended by the Echocardiographic Society (12). LV ejection fraction (LVEF) was assessed using the biplane modified Simpson method. All tests were conducted by experienced sonographers. Before patient recruitment, echocardiographic images of randomly sampled patients were collected from participating sites and were blindly reviewed and verified for diagnostic accuracy and measurement consistency at the core lab of Fuwai Hospital.

### Therapeutic Decisions

The therapeutic decisions were at the discretion of the attending practitioners and the patients. Patients were categorized according to the initial therapeutic decisions at the time of the index evaluation: (1) **Intervention group**: patients who were evaluated as suitable for surgical intervention and accepted the treatment proposal; (2) **Patient-refusal group**: patients who were evaluated as suitable for surgical intervention but refused the treatment proposal due to subjective preferences; (3) **Physician-deny group**: patients who were evaluated as unsuitable for surgical intervention and thereby were denied intervention by their attending practitioners.

### Clinical Outcomes

The primary outcome was 12-month all-cause mortality. Outcome data were obtained from medical records, patients visits, or telephone interviews. Death and intervention reports were validated by investigators at each center. On-site audits for source data verification were randomly administered in sample sites during the study implementation.

### Statistical Analyses

Continuous data with normal and non-normal distribution were expressed as mean ± standard deviation (SD) and median with interquartile range (IQR), respectively, and were compared using the Student *t*-test or Mann-Whitney *U*-test. Categorical data were presented as percentages and compared using the chi-square test. *P*-values were adjusted by the Benjamini-Hochberg method to control the false discovery rate in the multiple comparisons. Missing values were imputed using multiple imputations (Supplementary Table 2).

To identify the factors associated with physicians' decisions against intervention, we first used univariable logistic models to examine all baseline characteristics (Supplementary Table 3). Variables with *P* < 0.25 or clinical relevance were subsequently entered in the least absolute shrinkage and selection operator (LASSO) penalized logistic regression model (using “glmnet” R package), an improved statistical machine learning method that produces models with better performance than those produced by the stepwise selection methods widely used in the previous related articles (13). To maximize the predictive power and avoid overfitting of the model, the tuning parameter λ selection in the LASSO model used 10-fold cross-validation via minimum criteria. The Hosmer-Lemeshow goodness-of-fit test and C-index were used to assess the calibration and discrimination of the logistic model. In this analysis, patients in the intervention group and the patient-refusal group were combined and regarded as the patients for whom physicians decided to intervene, as both groups were assessed as suitable candidates and were proposed for intervention by physicians.

Predictors associated with 12-month mortality were initially analyzed using univariable Cox proportional hazards models. Variables with *P* < 0.25 or clinical relevance were then submitted to the LASSO-penalized Cox regression model for variable selection with the same tuning parameter λ selection approach abovementioned (Supplementary Table 4). Calibration and discrimination of the Cox model were examined with the Gronnesby-Borgan goodness-of-fit test and Harrell's C-index.

Survival analyses were performed to assess the prognostic impact of the initial therapeutic decisions, where all patients were included and patient categorization was based on the therapeutic decisions at the time of the index evaluation. Twelve-month survival distributions were visualized using the Kaplan-Meier method. We also calculated the expected survival of the enrolled patients based on the data of age- and sex-specific annual mortality rates of the Chinese general population (14). Cox proportional hazards models were performed to assess hazard ratios (HRs) with 95% confidence intervals (CIs). The models used inverse probability weighting (IPW) to reduce bias due to non-random decision assignment (15). The propensity scores used for IPW were estimated using multivariate logistic models where the therapeutic decision was the dependent variable, and plausible correlates of either the therapeutic decision-making or survival acted as independent variables. Based on the findings of our study and previous researches (7, 8, 16), the following variables were included: age, sex, body mass index (BMI), coronary heart disease (CHD), prior percutaneous coronary intervention (PCI), prior coronary artery bypass grafting (CABG), atrial fibrillation (AF), renal insufficiency, NYHA class III/IV, LVEF, CCI, and EuroSCORE-II. We also conducted subgroup analyses stratified by variables of interest that might exert a significant impact on 1-year survival, including advanced age (≥75-year-old and <75-year-old), severe comorbidities (CCI ≥ 5 and CCI <5), severe symptoms (NYHA III/IV and NYHA II), and reduced LVEF (LVEF <50% and LVEF ≥ 50%), with tests for interaction. Balance of covariates for adjustment before and after IPW adjustment was assessed using standardized mean difference plots (Supplementary Figure 2). The proportional hazard assumptions were verified by inspection of Schoenfeld residuals (Supplementary Figure 3). Statistical significance was set at 2-tail *P* < 0.05. All analyses were performed using R 4.0.2 (R Foundation for Statistical Computing, Vienna, Austria).

## Results

### Patients Characteristics, Therapeutic Decisions and Intervention

Patient characteristics are presented in Table 1. Of the 456 elderly patients with severe symptomatic AS included in this study, the median age was 69 years (IQR: 64–75), and 261 (57.2%) patients were male. Degenerative valve disease was the most frequent etiology (67.3%), followed by rheumatic valve disease (14.9%). Three hundred fifty five (77.9%) patients accepted the intervention proposal. Fifty two (11.4%) patients were proposed for intervention but refused, among whom 92.3% (48/52) were due to fear or reluctance of surgical intervention and 7.7% (4/52) were because of affordability concerns. Forty nine (10.7%) patients were denied intervention by their physicians after index evaluation.

**Table 1:** Baseline characteristics.

**Characteristic**	**Intervention group (*n* = 355)**	**Patient-refusal group (*n* = 52)**	**Physician-deny group (*n* = 49)**	Adjusted *P*-value (intervention vs. patient-refusal)^*^	Adjusted *P*-value (intervention vs. physician-deny)^*^
**Demographics**					
Age, yr [Median (IQR)]	68 (64–74)	72 (63–75)	76 (67–81)	0.153	<0.001
Male, no. (%)	199 (56.1%)	32 (61.5%)	30 (61.2%)	0.908	0.492
BMI, kg/m2 (Mean±SD)	23.68 ± 3.43	22.95 ± 3.74	23.85 ± 4.06	0.320	0.755
**Risk factors**					
Current Smoker, no. (%)	47 (13.2%)	5 (9.6%)	5 (10.2%)	0.900	0.541
Hypertension, no. (%)	171 (48.2%)	23 (44.2%)	29 (59.2%)	0.595	0.294
Diabetes, no. (%)	58 (16.3%)	6 (11.5%)	17 (34.7%)	0.358	0.008
Dyslipidemia, no. (%)	53 (14.9%)	4 (7.7%)	8 (16.3%)	0.402	0.800
**Comorbidities**					
Coronary heart disease, no. (%)	71 (20.0%)	16 (30.8%)	17 (34.7%)	0.088	0.052
Myocardial infarction, no. (%)	10 (2.8%)	0 (0%)	2 (4.1%)	0.192	0.646
Prior PCI, no. (%)	17 (4.8%)	6 (11.5%)	4 (8.2%)	0.152	0.350
Prior CABG, no. (%)	2 (0.6%)	1 (1.9%)	1 (2.0%)	0.337	0.644
Atrial fibrillation, no. (%)	33 (9.3%)	8 (15.4%)	8 (16.3%)	0.197	0.304
Cardiomyopathy, no. (%)	1 (0.3%)	1 (1.9%)	0 (0%)	0.478	0.611
Aortic disease, no. (%)	52 (14.6%)	3 (5.8%)	9 (18.4%)	0.170	0.506
Cerebrovascular disease, no. (%)^†^	31 (8.7%)	3 (5.8%)	5 (10.2%)	1.000	0.788
Peripheral artery disease, no. (%)	23 (6.5%)	1 (1.9%)	4 (8.2%)	0.680	0.554
COPD, no. (%)	14 (3.9%)	4 (7.7%)	3 (6.1%)	0.534	0.446
Renal insufficiency, no. (%)^‡^	59 (16.6%)	10 (19.2%)	16 (32.7%)	0.644	0.022
Malignant tumor, no. (%)	9 (2.5%)	1 (1.9%)	3 (6.1%)	0.783	0.336
Charlson comorbidity index, no. (%)				0.933	<0.001
1–2	11 (3.1%)	1 (1.9%)	1 (2.0%)		
2–4	201 (56.6%)	30 (57.7%)	11 (22.4%)		
≥5	143 (40.3%)	21 (40.4%)	37 (75.5%)		
**Symptoms**					
Angina pectoris, no. (%)	88 (24.8%)	19 (36.5%)	18 (36.7%)	0.162	0.084
NYHA class, no. (%)				0.042	<0.001
II	116 (32.7%)	16 (30.8%)	13 (26.5%)		
III	180 (50.7%)	21 (40.4%)	16 (32.7%)		
IV	37 (10.4%)	13 (25.0%)	18 (36.7%)		
Syncope, no. (%)	37 (10.4%)	2 (3.8%)	9 (18.4%)	0.204	0.246
**Investigations**					
LVEF, no. (%)				0.034	<0.001
>50%	285 (80.3%)	33 (63.5%)	24 (49.0%)		
30–50%	59 (16.6%)	16 (30.8%)	18 (36.7%)		
≤ 30%	11 (3.1%)	3 (5.8%)	7 (14.3%)		
LV, mm [Median (IQR)]	51 (46–55)	52 (48–57)	54 (48–59)	0.054	0.066
LA, mm [Median (IQR)]	41 (37–46)	43 (37–49)	43 (40–50)	0.097	0.038
Combined moderate AR, no.(%)	103 (29%)	14 (26.9%)	9 (18.4%)	0.754	0.212
Combined moderate MVHD, no.(%)^§^	67 (18.9%)	15 (28.8%)	15 (30.6%)	0.107	0.134
Pulmonary hypertension, no.(%)	85 (23.9%)	8 (15.4%)	18 (36.7%)	0.154	0.126
**EuroSCORE-II** [Median (IQR)]	2.0 (1.3–3.8)	2.3 (1.4–3.4)	9.1 (5.1–11.9)	0.617	<0.001

*y, year; no., number; BMI, body mass index; PCI, percutaneous coronary intervention; CABG, coronary artery bypass grafting; COPD, chronic obstructive pulmonary disease; NYHA, New York heart association class; LVEF, left ventricular ejection fraction; LV, left ventricular end-diastolic dimension; LA, left atrium end-diastolic dimension; AR, aortic regurgitation; MVHD, multiple valvular heart disease*.

* *P-values were adjusted by the Benjamini-Hochberg method to control the false discovery rate in the multiple comparisons*.

† *Cerebrovascular disease was defined as the history of neurologic deficit syndrome caused by ischemia or hemorrhage*.

‡ *Renal insufficiency was defined as estimated glomerular filtration rate (eGFR) <60 ml/min/1.73 m^2^*.

§ *In addition to aortic valvular lesion, one or more of the mitral, tricuspid and pulmonary valves were with stenosis or regurgitation lesions*.

Of the patients who accepted the intervention proposal, 290 (81.7%) patients underwent immediate intervention at the participating centers during the enrollment period, among whom 88.6% (257/290) underwent surgical AVR, while 9.3% (27/290) received TAVR (Table 2). Sixty five (18.3%) patients were scheduled for elective intervention; at 12-month follow-up, 21 patients on the waiting list received intervention and 19 patients died before the operation. Notably, 8 (15.4%) patients who initially refused the intervention proposal underwent the subsequent intervention during follow-up, but with an average delay of 195 days.

**Table 2:** Intervention and medication use.

**Characteristic**	**Intervention group (*n* = 355)**	**Patient-refusal group (*n* = 52)**	**Physician-deny group (*n* = 49)**	Adjusted *P*-value (intervention vs. patient-refusal)^*^	Adjusted *P*-value (intervention vs. physician-deny)^*^
**Immediate intervention**	290 (81.7%)	0 (0%)	0 (0%)	<0.001	<0.001
SAVR, no. (%)	257 (72.4%)	0 (0%)	0 (0%)	<0.001	<0.001
TAVR, no. (%)	27 (7.6%)	0 (0%)	0 (0%)	0.035	0.060
Balloon aortic valvuloplasty, no. (%)	6 (1.7%)	0 (0%)	0 (0%)	0.610	0.615
Concomitant operation					
CABG, no. (%)	31 (8.7%)	0 (0%)	0 (0%)	0.022	0.022
Aortic surgery, no. (%)	37 (10.4%)	0 (0%)	0 (0%)	0.008	0.014
**Intervention during follow-up**^†^	21 (5.9%)	8 (15.4%)	1 (2.0%)	0.026	0.497
SAVR, no. (%)	13 (3.7%)	7 (13.5%)	0 (0%)	0.008	0.382
TAVR, no. (%)	8 (2.3%)	0 (0%)	1 (2.0%)	0.401	0.924
Balloon aortic valvuloplasty, no. (%)	0 (0%)	1 (1.9%)	0 (0%)	0.128	1.000
**Medication use at discharge**					
Warfarin, no. (%)	234 (65.9%)	6 (11.5%)	7 (14.3%)	<0.001	<0.001
Antiplatelet agents, no. (%)^‡^	122 (34.4%)	22 (42.3%)	28 (57.1%)	0.268	0.004
Diuretics, no. (%)	320 (90.1%)	35 (67.3%)	42 (85.7%)	<0.001	0.361
β receptor inhibitor	209 (58.9%)	16 (30.8%)	32 (65.3%)	<0.001	0.386
ACEI/ARB, no. (%)	88 (24.8%)	9 (17.3%)	21 (42.9%)	0.223	0.020
Digitalis, no. (%)	97 (27.3%)	15 (28.8%)	11 (22.4%)	0.819	0.926

*no., number; SAVR, surgical aortic valve replacement; TAVR, transcatheter aortic valve replacement; CABG, coronary artery bypass grafting; ACEI, angiotensin-converting enzyme inhibitors; ARB, angiotensin receptor blocker*.

* *P-values were adjusted by the Benjamini-Hochberg method to control the false discovery rate in the multiple comparisons*.

† *Patients who had received surgical intervention during hospitalization and underwent re-operation during follow-up were not included in the count*.

‡ *Antiplatelet drugs included aspirin, P2Y12 receptor inhibitor, cilostazol and persantin*.

### Analysis of Decisions Against Intervention

Patient characteristics associated with physicians' decisions against intervention in the univariable analysis are summarized in Supplementary Table 3. Surgical intervention was more frequently denied by physicians in those who were older and had diabetes, CHD, renal insufficiency, larger LA dimension, and worse cardiac function as attested by higher NYHA classes and reduced LVEF. Correspondingly, the CCI and EuroSCORE-II were also higher in these patients (All *P* < 0.05).

**Figure 2:**
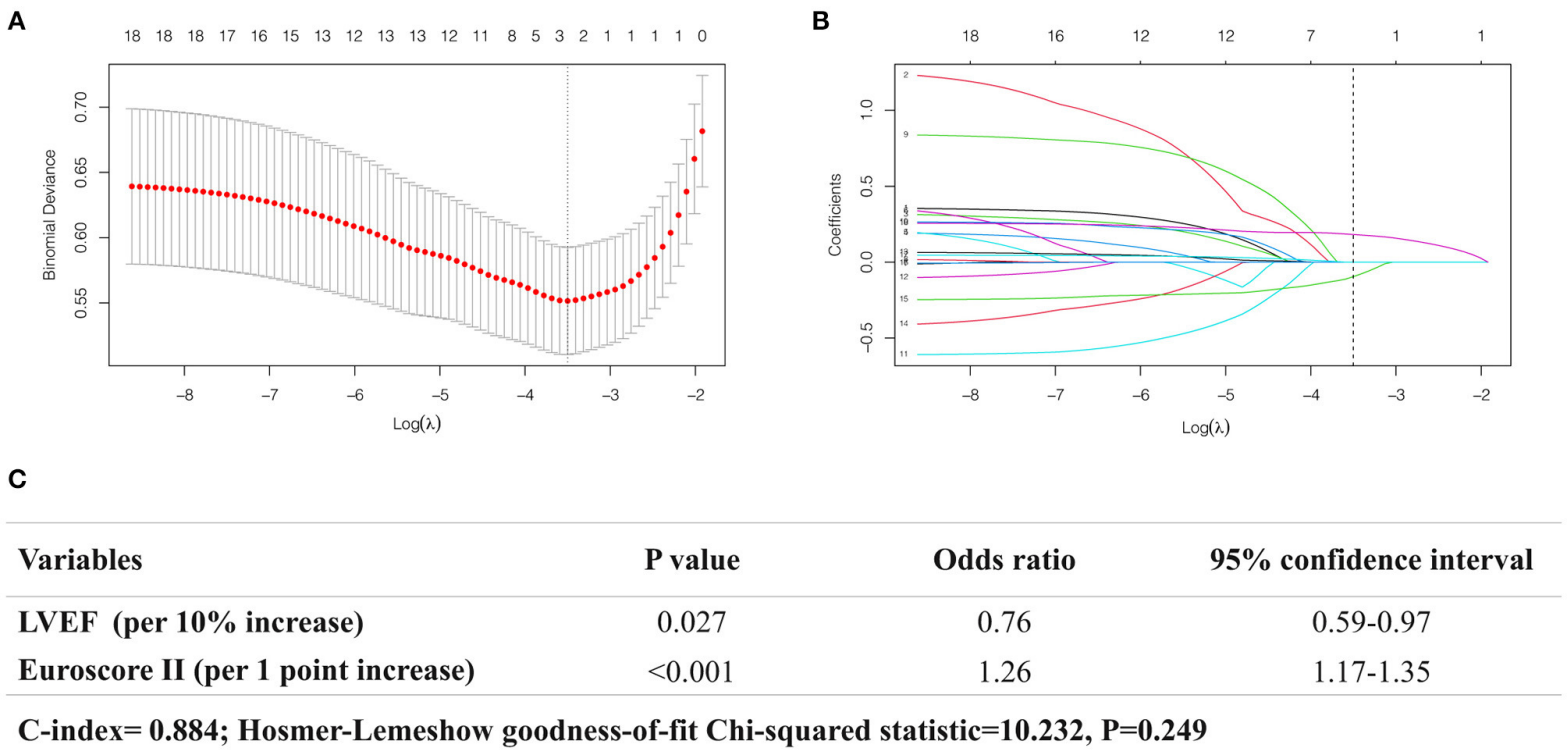
Analysis of the factors associated with physicians' decisions against intervention using LASSO-penalized logistic regression model. **(A)** The plot showing the deviance values of the LASSO model as a function of the tuning parameter λ. The optimal λ is the value that minimizes the deviance curve (dashed line). **(B)** Trace plot showing non-zero model coefficients as a function of the tuning parameter λ. As the λ increases, LASSO sets various coefficients to zero, thus removing them from the model. When λ corresponds to the minimum-deviance (dashed line), two variables are selected **(C)**.

In multivariable analysis, the two factors significantly associated with physicians' decisions against intervention selected by the LASSO-penalized logistic model were reduced LVEF (per 10% increase, Odds ratio (OR): 0.76; 95% CI: 0.59–0.97; *P* = 0.027) and increased EuroSCORE-II (per 1 point increase, OR 1.26; 95% CI: 1.17–1.35; *P* < 0.001) (Figure 2). The model achieved good discrimination and calibration, with C-index at 0.884 and Hosmer-Lemeshow goodness-of-fit test *P* = 0.249.

### Analysis of 12-Month Outcome

The 12-month follow-up was available in 386 patients. Death occurred in 49 patients: 22 (6.5%) in the intervention group, 9 (18.5%) in the patient-refusal group, and 18 (39.6%) in the physician-deny group.

In univariable analysis (Supplementary Table 4), patients' decisions to refuse intervention and physicians' decisions to deny intervention were both associated with increased mortality risk. Besides, the risk of death was also higher in patients who were older and had diabetes, AF, cerebrovascular disease, renal insufficiency, syncope, reduced LVEF, larger LV and LA dimension, combined multiple valvular heart disease, and increased CCI and EuroSCORE-II (All *P* < 0.05).

**Figure 3:**
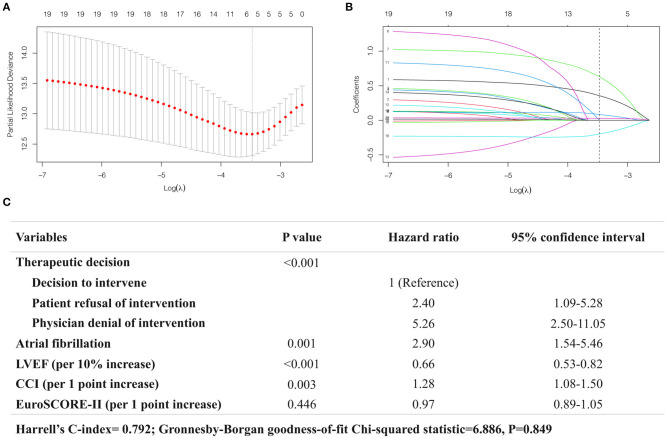
Analysis of the predictors of 12-month mortality using LASSO-penalized Cox regression model. **(A)** The plot showing the deviance values of the LASSO model as a function of the tuning parameter λ. The optimal λ is the value that minimizes the deviance curve (dashed line). **(B)** Trace plot showing non-zero model coefficients as a function of the tuning parameter λ. As the λ increases, LASSO sets various coefficients to zero, thus removing them from the model. When λ corresponds to the minimum-deviance (dashed line), five variables are selected **(C)**.

After variable selection using LASSO-penalized Cox regression model, the five predictors strongly associated with 12-month mortality were patients' decisions to refuse intervention (HR: 2.40; 95% CI: 1.09–5.28; *P* < 0.001), physicians' decisions to deny intervention (HR: 5.26; 95% CI: 2.50–11.05; *P* < 0.001), AF (HR: 2.90; 95% CI: 1.54–5.46; *P* = 0.001), reduced LVEF (per 10% increase, HR: 0.66; 95% CI: 0.53–0.82; *P* < 0.001), increased CCI (per one point increase, HR: 1.28; 95% CI: 1.08–1.50; *P* = 0.003). EuroSCORE-II was also selected into the model but did not reach the significance level (*P* = 0.446) (Figure 3). The model presented a good predictive power, with Harrell's C-index at 0.792 and Gronnesby-Borgan goodness-of-fit test *P* = 0.849.

### Prognostic Impact of Therapeutic Decisions

**Figure 4:**
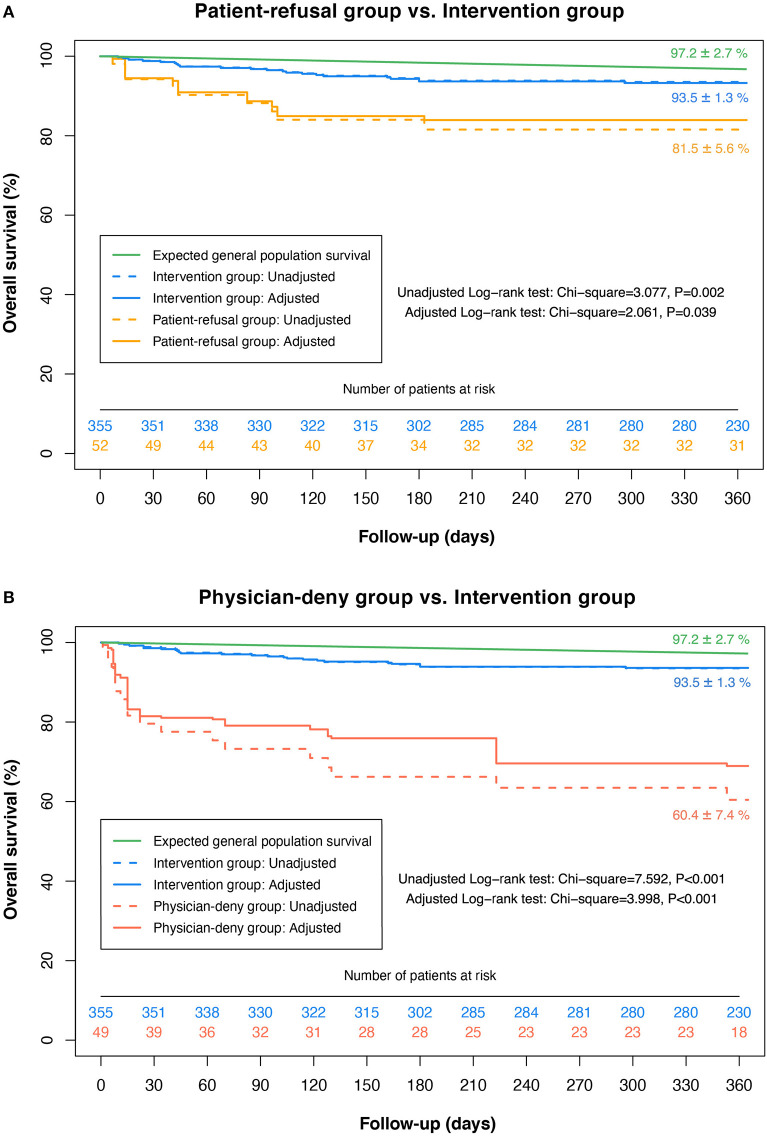
Kaplan-Meier survival curves for patients with different therapeutic decisions. The plot showing the survival distributions of patients who accepted the intervention proposal (Blue); refused the intervention proposal (Yellow); were denied intervention by physicians (Red), as well as the expected survival of the age- and sex-specific general population (Green). The dash lines represent the crude survival rates and the solid lines indicate the adjusted survival rates using IPW.

Figure 4 depicts the crude and adjusted Kaplan-Meier curves for survival between the three groups, together with the expected survival in the age- and sex-specific Chinese general population. At 12 months, there was a significant survival benefit in favor of the intervention group (93.5 ± 1.3%) as compared to the patient-refusal group (81.5 ± 5.6%, log-rank *P* = 0.002) and the physician-deny group (60.4 ±7.4%, log-rank *P* < 0.001). After IPW adjustment (Table 3), the decisions against intervention made by patients or by physicians were both significantly associated with 12-month mortality, and physician's denial decision was a stronger determinant of death (HR: 7.30; 95%CI: 3.35–15.92; *P* < 0.001) than patient's refusal decision (HR: 2.61; 95%CI: 1.09–6.20; *P* = 0.031). In subgroup analyses, the results remained consistent with the overall findings, and there were no significant interactions between the therapeutic decisions and subgroup variables for 1-year mortality (all *P*-interaction > 0.05) (Table 3).

**Table 3:** Association of therapeutic decisions with 12-month mortality.

**Study group**	**Therapeutic decisions**	**Univariate analysis of 12-month mortality**		**Multivariate analysis of 12-month mortality using IPW**^**†**^		***P* for interaction:**
		**Crude HR (95% CI)**	***P*-value**	**Adjusted HR (95% CI)**	***P*-value**	
Whole cohort	Patient-refusal vs. Intervention	3.17 (1.46–6.89)	0.004	2.61 (1.09–6.20)	0.031	
	Physician-denial vs. Intervention	7.74 (4.15–14.45)	<0.001	7.30 (3.35–15.92)	<0.001	
Age ≥ 75	Patient-refusal vs. Intervention	2.22 (0.45–11.05)	0.329	4.85 (1.10–21.43)	0.037	Decisions*Age: *P* = 0.529
	Physician-denial vs. Intervention	8.47 (3.16–22.65)	<0.001	11.99 (3.79–37.94)	<0.001	
Age <75	Patient-refusal vs. Intervention	4.08 (1.74–9.53)	0.001	2.76 (1.07–7.11)	0.036	
	Physician-denial vs. Intervention	5.80 (2.27–14.83)	<0.001	8.61 (3.29–22.52)	<0.001	
CCI ≥ 5	Patient-refusal vs. Intervention	5.37 (1.27–22.65)	0.022	5.02 (1.21–20.83)	0.026	Decisions*CCI: *P* = 0.282
	Physician-denial vs. Intervention	6.35 (3.05–13.24)	<0.001	5.90 (2.51–13.87)	<0.001	
CCI <5	Patient-refusal vs. Intervention	4.20 (1.41–12.53)	0.010	3.54 (1.09–11.53)	0.036	
	Physician-denial vs. Intervention	4.72 (1.02–21.85)	0.047	11.70 (3.11–44.09)	<0.001	
NYHA III/IV	Patient-refusal vs. Intervention	3.16 (1.21–8.22)	0.019	2.86 (1.03–7.93)	0.043	Decisions*NYHA: *P* = 0.147
	Physician-denial vs. Intervention	9.93 (4.77–20.66)	<0.001	7.85 (3.30–18.69)	<0.001	
NYHA II	Patient-refusal vs. Intervention	4.69 (1.12–19.62)	0.035	5.08 (1.18–21.77)	0.029	
	Physician-denial vs. Intervention	5.65 (1.35–23.63)	0.018	6.35 (1.38-29.09)	0.017	
LVEF <50%	Patient-refusal vs. Intervention	4.40 (1.33–14.54)	0.015	5.47 (1.32–22.63)	0.019	Decisions*LVEF: *P* = 0.537
	Physician-denial vs. Intervention	7.11 (2.62–19.29)	<0.001	6.12 (2.01–18.58)	0.001	
LVEF≥50%	Patient-refusal vs. Intervention	2.73 (1.00–7.45)	0.050	3.60 (1.08–12.05)	0.038	
	Physician-denial vs. Intervention	6.07 (2.50–14.77)	<0.001	5.42 (1.76–16.66)	0.003	

*CCI, Charlson comorbidity index; NYHA, New York heart association class; LVEF, left ventricular ejection fraction; HR, hazard ratio; CI, confidence interval*.

† *Adjusted for age, sex, body mass index, coronary heart disease, prior percutaneous coronary intervention, prior coronary artery bypass grafting, atrial fibrillation, renal insufficiency, New York Heart Association class III/IV, left ventricular ejection fraction, Charlson Comorbidity Index, and EuroSCORE-II*.

## Discussion

In this prospective cohort study detailing contemporary therapeutic decision-making and outcomes of elderly patients with severe symptomatic AS in a wide range of centers throughout mainland China, our key findings are as follows: (1) In current clinical practice, the decision against intervention was taken in about one-fifth of elderly patients with symptomatic severe AS, half of which were due to patients' subjective preferences. (2) Reduced LVEF and increased EuroSCORE-II were strongly associated with physicians' decisions to deny intervention, suggesting that surgical risk remained the primary concern for physicians when making therapeutic decisions. (3) Elderly patients' initial decisions against intervention profoundly impacted subsequent intervention rates and prognosis. Only 15.4% of patients who initially refused the intervention proposal underwent the subsequent intervention within a year, with a noticeable delay in the timing of procedures.

### Surgical Intervention in Elderly Patients With AS

Previous studies reported that surgical intervention was decided against in 33–41% of elderly patients with severe symptomatic AS (6–8). However, the corresponding figure in the present study was much lower at 22.1%. The reasons for this improvement are multifactorial. First, enhancement in surgical, anesthetic, and intensive-care techniques over the past decades certainly play a key role in facilitating more aggressive treatment strategies in these elderly patients. Second, the advent of the new catheter techniques, TAVR, has allowed patients who were deemed at high or prohibitive surgical risk to undergo AVR procedures (3, 4). Third, it can be speculated that patient acceptance of invasive intervention may have increased during the last few decades, given our observation that patients' subjective preferences almost accounted for half of the decisions against intervention.

### Factors Associated With Physicians' Decisions Against Intervention

To investigate why elderly patients with severe AS were denied intervention by their attending practitioners, we chose to analyze the objective characteristics of patients rather than the reasons given by physicians, in order to limit the subjective component in patient evaluation. We found that reduced LVEF and increased EuroSCORE-II were strongly associated with physicians' decisions to deny intervention in current clinical practice. Reduced LVEF has been widely acknowledged as a strong predictor of operative mortality in cardiovascular surgery (11, 17). Similarly, EuroSCORE-II is a multivariable scoring system designed to estimate thoracic surgical risk with excellent predictive power (18). These two selected factors suggest that surgical risk may be the primary consideration for physicians when making therapeutic decisions for elderly patients with severe AS. However, the increase in surgical risk associated with reduced LVEF is most prominent in patients with severe ventricular dysfunction, that is, LVEF <30%, which accounted for only a small proportion of our study population. Conversely, patients with ventricular dysfunction could benefit substantially from surgery (6, 19), which was supported by our findings that the decision to intervene was strongly associated with improved survival in patients with or without ventricular dysfunction, and there was no significant interaction between reduced LVEF and therapeutic decisions for mortality risk. Therefore, the denial of intervention solely based on reduced LVEF, particularly LVEF between 30 and 50%, is neither substantiated by the available evidence nor supported by guidelines (3, 4).

In previous articles, advanced age was also identified as a determinant factor of the decision against intervention (7, 8). Advanced age is indeed associated with increased surgical risk in cardiovascular surgery, particularly in AS cases (17, 20, 21). However, it has been shown that elderly patients with AS could derive a particular survival benefit from AVR, with an acceptable mortality risk compared to the expected survival of the age-matched general population (5, 8, 22). These findings have led to the current guidelines recommendation that AVR should not be denied on the sole grounds of advanced age (3, 4). In our study, the association of age with physicians' decisions to deny intervention was not significant, which may be attributed to improved compliance with the guideline-recommended management in contemporary clinical practice.

### Prognostic Impact of Patients' Initial Decisions Against Intervention

In general, therapeutic decisions are made by consensus between patients and physicians. After assessing the risk-benefits ratio, attending practitioners will provide intervention proposals to suitable patients, while the final decision to accept it or not is at the discretion of patients. Refusal of intervention is frequent in the elderly population, but lacks sufficient attention. It has been shown that elderly patients with severe AS were more likely to opt for conservative treatment than younger patients, and the most common reasons cited for the refusal decisions were fear of surgical complications and unawareness of prognostic information (23, 24). However, the prognostic impact of patients' initial decisions against intervention has not yet been convincingly demonstrated in the literature, as the previous articles seldom distinguished whether the decisions against intervention were made by patients or by their attending practitioners.

In the present study, we observed that, despite having developed symptoms, only 15.4% of patients who initially refused the intervention proposal underwent the subsequent intervention at 12-month follow-up, with a noticeable delay of over 6 months on average. This suggests that elderly patients seem to be obstinate in their initial decisions and less likely to actively seek intervention if the refusal decisions have already been made. Although patients who refused intervention due to subjective preferences presented similar risk profiles to those who accepted the intervention proposal, the limited intervention rate and delayed treatment still resulted in a dismal prognosis. The 12-month survival rate of the patient-refusal group was only 81.5%, contrasting sharply with that of the intervention group (93.5%). Correspondingly, the refusal decision made by patients was identified as an independent predictor of 12-month mortality.

Thus, elderly patients' initial decisions against intervention should be treated as a “risk factor” at the subjective level, given its significant impact on subsequent treatment and strong association with mortality. More efforts are necessary to provide patient education concerning the actual risks and survival benefits of the AVR procedure and motivate the suitable patients to accept the intervention proposal during the medical contact. For those who have refused intervention, closer follow-up along with intensive patient education is warranted.

### Implication for Patients Who Were Denied Intervention at the TAVR Era

It was notable that patients who were denied intervention by physicians had the worst prognosis among the three groups, with only 60.4% of them surviving to 12 months. These patients tended to have worse clinical profiles, characterized by advanced age, more comorbidities as evidenced by increased CCI, and impaired cardiac function with reduced LVEF. Comorbidities and heart failure are frequent in the elderly population and directly influence life expectancy regardless of the valvular heart disease (25–27), which is evidenced by the substantial predictive value of AF, CCI, and LVEF for 12-month mortality in our study. This explains why their prognosis was also inferior to that of the patient-refusal group with a similarly limited intervention rate. However, even after adjusting for these potential confounders using IPW, the remarkable disparity in mortality between the intervention group and the physician-denial group persisted, indicating the decisive role of surgical intervention for prognosis.

In recent years, the development of TAVR has led to a major paradigm shift in the management of AS and extended AVR procedure to previously undertreated patients with high or prohibitive surgical risk (3, 4). Recent investigations in Europe and the US reported that TAVR had constituted almost half of AVR procedures (28, 29). However, TAVR was commercially available in China after 2017, 5–10 years later than the western countries (30). In this nationwide survey conducted at the beginning of the TAVR era in China, TAVR accounted for only 11.3% (36/319) of total AVR procedures, and thus surgery remained the primary treatment for AS. As TAVR becomes more widely available, it is predictable that more patients who cannot undergo surgery will benefit from it.

Some limitations should be acknowledged in the present study. First, the bias of hospital selection may exist in the present study. Although the participating centers were all large academic hospitals with a wide geographical distribution covering most provinces and municipalities throughout China, these hospitals were not selected at random, which may reduce the epidemiological representativeness of our results. Second, as an observational study, therapeutic decisions were not randomly assigned, which may introduce bias into the analyses of the prognostic impact of therapeutic decisions. Although we used IPW to reduce the bias inherent to the observational study, the possibility of unmeasured confounding factors still exists. Third, angiographically significant coronary artery disease may be a potential confounder that increases the surgical risk, but its weight in therapeutic decision-making cannot be assessed, as the performance of angiography is closely linked with the decision to intervene (3, 4). This leads to bias in the evaluation of the prevalence of coronary artery disease in patients with the decisions against intervention. Moreover, since coronary angiography was not performed in all cases, the Society of Thoracic Surgeons (STS) score cannot be calculated. Therefore, we chose to describe surgical risk using EuroSCORE-II. These two scoring systems have been proven to be equivalently effective with comparable discrimination concerning AVR outcomes (18). Fourth, the China-DVD cohort study was designed to follow up for only 1 year. However, despite the relatively short follow-up period, the survival rates had shown prominent disparities among patients with different therapeutic decisions, with sufficient statistical significance to support our hypothesis.

In conclusion, this nationwide prospective study shows that surgical intervention was decided against in 22.1% of elderly patients with severe symptomatic AS, half of which were due to patients' subjective preferences. Surgical risk remains the primary concern for physicians when making therapeutic decisions, whereas the weight of reduced LVEF in the decisions against intervention appears to be unjustified based on current knowledge. Patients who initially refused the intervention proposal rarely seek intervention in time after discharge, resulting in a dismal prognosis. Thus, elderly patients' initial decisions against intervention should be treated as a “risk factor” at the subjective level, which has been neglected in clinical practice. To further improve the management of elderly patients with AS, efforts should be made to enhance patient acceptance of intervention via intensive patient education and increase the intervention rates for patients with high or prohibitive surgical risk by utilizing the TAVR technique.

## Data Availability Statement

The raw data supporting the conclusions of this article will be made available by the authors, without undue reservation.

## Ethics Statement

The studies involving human participants were reviewed and approved by Institution Review Board central committee at Fuwai Hospital, National Center for Cardiovascular Diseases of China. The patients/participants provided their written informed consent to participate in this study.

## Author Contributions

YW and HX conceived the study. QZ, HX, and BZ developed the study methodology. QZ and QL conducted statistical analyses. QZ edited the initial draft of the manuscript. YY, ZL, RG, and YW critically revised the manuscript for important intellectual content. All authors contributed to the article and approved the submitted version.

## Conflict of Interest

The authors declare that the research was conducted in the absence of any commercial or financial relationships that could be construed as a potential conflict of interest.

## Publisher's Note

All claims expressed in this article are solely those of the authors and do not necessarily represent those of their affiliated organizations, or those of the publisher, the editors and the reviewers. Any product that may be evaluated in this article, or claim that may be made by its manufacturer, is not guaranteed or endorsed by the publisher.

## Abbreviations

AS: aortic stenosis
AVR: aortic valve replacement
TAVR: transcatheter aortic valve replacement
VHD: valvular heart disease
NYHA: New York Heart Association
CCI: Charlson comorbidity index
EuroSCORE: European system for cardiac operative risk evaluation
LV: left ventricles
LA: left atrium
LVEF: left ventricular ejection fraction
LASSO: the least absolute shrinkage and selection operator
IPW: inverse probability weighting
PCI: percutaneous coronary intervention
CABG: coronary artery bypass grafting
BMI: body mass index
AF: atrial fibrillation
COPD: chronic obstructive pulmonary disease
ACEI: angiotensin-converting enzyme inhibitors
ARB: angiotensin receptor blocker.
