# Supplementary material for: Therapeutic Decision-Making and Outcomes in Elderly Patients With Severe Symptomatic Aortic Stenosis: Prognostic Implications of Elderly Patients' Initial Decisions
Source: Front Cardiovasc Med. 2021 Jul 26;8:696763. doi: 10.3389/fcvm.2021.696763 (PMC8350052; doi:10.3389/fcvm.2021.696763)
Supplement: Supplementary file 1 [file Data_Sheet_1.docx]

**SUPPLEMENTARY MATERIAL**

Zhao et al. Therapeutic decision-making and outcomes in elderly patients with severe symptomatic aortic stenosis: prognostic implications of elderly patients’ initial decisions

**Contents:**

**Supplementary methods**

**Diagnostic criteria of moderate and severe native VHD**

**Data collection, management, and quality control of China-DVD study**

**Supplementary Tables**

**Supplementary Table 1. Hospitals participating in the China-DVD study**

**Supplementary Table 2. Number of missing values and corresponding dispositions**

**Supplementary Table 3: Univariable analysis of the factors associated with physicians’ decisions against intervention**

**Supplementary Table 4: Univariable analysis of the predictive factors of 12-month mortality**

**Supplementary Table 5: Comparison of the multivariate models of physicians’ decisions against intervention generated using the stepwise selection method and the LASSO method**

**Supplementary Table 6: Comparison of the multivariate models of 12-month mortality generated using the stepwise selection method and the LASSO method**

**Supplementary Table 7: Standardized mean differences across covariates before and after IPW adjustment.**

**Supplementary Table 8: Sensitivity analysis of patients whose actual treatments in line with their initial therapeutic decisions**

**Supplementary Figure**

**Supplementary Figure 1. Geographical distribution of hospital participating in the China-DVD study**

**Supplementary Figure 2. Absolute standardized mean differences across covariates before and after IPW adjustment.**

**Supplementary Figure 3. ﻿Graphical inspection of scaled Schoenfeld residuals to test the proportional hazard assumption for 12-month mortality.**

**Supplementary methods**

**Diagnostic criteria of moderate and severe native VHD**

Detailed criteria of significant native valvular heart disease (VHD) in the China-DVD study were defined following the 2014 ACC/AHA guidelines: aortic stenosis (AS) with a valve area ≤1.5cm ^2^ or a maximal jet velocity ≥3m/s or pressure gradient ≥20mmHg, aortic regurgitation (AR) with a grade ≥2/4, mitral stenosis (MS) with a valve area ≤2.0cm^2^ , mitral regurgitation (MR) with a grade ≥2/4, pulmonary stenosis (PS), pulmonary regurgitation (PR), tricuspid stenosis (TS), tricuspid regurgitation (TR) with moderate or severe grade. Severe VHD was defined as AS with a valve area ≤1.0cm^2^ or a maximal jet velocity ≥4m/s or pressure gradient ≥40mmHg, AR with a regurgitation grade ≥3/4, MS with a valve area ≤1.5cm^2^, MR with a regurgitation grade ≥3/4, PS with a maximal jet velocity ≥4m/s, PR with the severe grade, TS with a valve area ≤1.0cm^2^ or pressure gradient ≥5mmHg, TR with severe grade.

**Data collection, management, and quality control**

All local investigators received detailed training on the protocols and data collection. A standard echocardiography protocol was provided to operators and reporters at participating sites.

On the first page of the case report form (CRF) was the inclusion criteria list to remind the investigators and make sure the accuracy of enrollment. Site investigators were required to collect the data during the hospitalization and complete all the information in CRF upon the patient’s discharge or death. Appointed and trained local cardiologists were responsible for the quality of data collection at their own hospitals. We also requested the sites to scan the medical records and echocardiography reports then sent them to the coordinator center of the study as resource documents for the check. Once all CRFs were sent to the coordinating center, the coordinators checked the inclusion criteria on the first page and the completeness of all variables. The CRFs with queries were sent back to the sites for correction and completion if the data were missing or not reasonable. All data were entered into electronic data capture (EDC) by trained medical students in a double parallel manner, subsequent comparison and recheck to make sure of the accordance and accuracy of data. Data managers performed multiple checks on the EDC dataset according to data management protocol and provided the quality reports. Data reports on the descriptions of all variables were performed to find the logistical problems and then search the source documents for check and correction. We first excluded the duplicate cases and the cases that did not meet the age criteria or valve disease criteria. Then the values excessively out of normal range and missing data were checked, corrected and completed based on source documents. Particularly, the important variables such as etiology, echocardiography, comorbidities were checked by the experienced clinical cardiologists at Fuwai Hospital, based on medical records and imaging reports.

Randomly sampled hospital sites received on-site audits. Trained auditors examined the understanding extent of the study protocol for local investigators. They went through discharge records of the hospitals during one randomly decided week within the enrollment period to evaluate the consecutiveness of enrollment. They also reviewed inpatient medical records of patients drawn randomly to check the accuracy of diagnosis and data and consistency with CRFs. In addition, echocardiography videos of a limited number of randomly sampled patients were reviewed and validated blindly at the core echocardiography lab at Fuwai Hospital.

**Supplementary statistical analyses**

To assess the incremental value of the multivariate models generated using the LASSO method and beyond the stepwise selection method in our study population, we employed a forward stepwise logistic regression procedure to select factors independently associated with the physicians’ decisions against intervention (**Supplementary Table 5**) and a forward stepwise Cox regression procedure to select independent predictors of 12-month mortality (**Supplementary Table 6**), where all baseline variables with P<0.25 or clinical relevance were entered. To avoid collinearity problems in the regression models, the correlation coefficients between covariates were examined. The performance of the multivariate models generated using the stepwise selection method was compared with those generated using the LASSO method as mentioned in the text. C-index, integrated discrimination improvement (IDI) index, and net reclassification improvement (NRI) index were calculated to evaluate the discrimination properties, and likelihood ratio (LR) test and Bayesian information criteria (BIC) were used to assess the calibration properties.

To further verify the consistency of our findings in patients whose actual treatments in line with their initial therapeutic decisions, we performed a sensitivity analysis in which patients who initially refused the intervention proposal or were denied intervention by their physicians were censored at the time of intervention if they received intervention during the follow-up; patients who initially accepted the intervention proposal were excluded if they did not receive the scheduled procedures during the follow-up or died before the operation. In this sensitivity analysis, hazard ratios (HRs) with 95% confidence intervals (CIs) were assessed using Cox proportional hazards model, with multivariate adjustment using inverse probability weighting (IPW).

**Supplementary Table 1: Hospitals participating in the China-DVD study**

| Hospital | Province | Local PI |
| --- | --- | --- |
| Fuwai Hospital | Beijing | Yongjian Wu, Haiyan Xu |
| Beijing Anzhen Hospital | Beijing | Wei Liu, Haibo Zhang |
| Chinese PLA General Hospital | Beijing | Changfu Liu |
| Tianjin First Central Hospital | Tianjing | Changzhi Lu |
| Taida International Cardiovascular Hospital | Tianjing | Zhigang Liu |
| Tianjin Chest Hospital | Tianjing | Hongliang Cong |
| Zhongshan Hospital affiliated to Fudan University | Shanghai | Daxin Zhou |
| Ruijin Hospital affiliated to Shanghai Jiaotong University | Shanghai | Ruiyan Zhang |
| Shanghai Changhai Hospital | Shanghai | Xianxian Zhao |
| Shanghai Changzheng Hospital | Shanghai | Chun Liang |
| Shanghai Chest Hospital | Shanghai | Xinkai Qu |
| Xinqiao Hospital | Chongqing | Shiyong Yu |
| Western-south Hospital | Chongqing | Zhihui Zhang |
| First Hospital affiliated to Haerbin Medical University | Heilongjiang | Yue Li |
| Second Hospital affiliated to Haerbin Medical University | Heilongjiang | Bo Yu |
| Second Hospital affiliated to Jilin University | Jilin | Bin Liu |
| Northern Hospital | Liaoning | Quanmin Jing |
| Zhongshan Hospital affiliated to Dalian Medical University | Liaoning | Xiaoming Bian |
| Inner Mongolia Medical University Hospital | Inner Mongolia | Yuexi Wang |
| Fisrt Hospital affiliated to Baotou Medical School | Inner Mongolia | Hanjun Pei |
| Shanxi Cardiovascular Hospital | Shanxi | Jian An |
| Shanxi Provincial Hospital | Shanxi | Hong Zhang |
| Xijing Hospital | Shaanxi | Lin Tao, Jian Yang |
| First Hospital affiliated to Xian Jiaotong University | Shaanxi | Zuyi Yuan |
| Tangdu Hospital affiliated to Forth Army Medical University | Shaanxi | Haichang Wang |
| First Hospital affiliated to Xinjiang Medical University | Xinjiang | Xiang Ma |
| Hospital affiliated to Qinghai University | Qinghai | Weijun Liu |
| Gansu Provincial Hospital | Gansu | Ping Xie |
| First Hospital affiliated to Lanzhou University | Gansu | Zheng Zhang |
| General Hospital affiliated to Ningxia Medical University | Ningxia | Saobin Jia |
| Linyi People Hospital | Shandong | Yanjin We |
| Hospital affiliated to Jining Medical School | Shandong | Qingxian Li |
| Qingdao Fuwai Hospital | Shandong | Xianyan Jiang |
| Yantai Yuhuangding Hospital | Shandong | Chuanhuan Zhang |
| Henan Provincial Hospital | Henan | Chuanyu Gao |
| First Hospital affiliated to Zhengzhou University | Henan | Chunguang Qiu |
| Zhengzhou Seventh People Hospital | Henan | Yiqiang Yuan |
| Nanjin First Hospital | Jiangsu | Shaoliang Chen |
| Nanjing Gulou Hospital | Jiangsu | Biao Xu |
| Xuzhou Central Hospital | Jiangsu | Bin Han |
| Wuhan Union Hospital | Hubei | Xiang Cheng |
| Asian Heart Disease Hospital | Hubei | Xi Su |
| Xiangya Hospital | Hunan | Zaixin Yu |
| Xiangya Second Hospital | Hunan | Xinqun Hu |
| Anhui Provincial Hospital | Anhui | Likun Ma |
| Yijishan Hospital affiliated to Wannan Medical School | Anhui | Yongshen Ke |
| Bengbu Medical School Hospital | Anhui | Hongju Wang |
| Jiangxi Provincial Hospital | Jiangxi | Lang Hong |
| Fist Hospital affiliated to Nanchang University | Jiangxi | Zheqi Zheng |
| Second Hospital affiliated to Nanchang University | Jiangxi | Yanqing Wu |
| First Hospital affiliated to Zhejiang University | Zhejiang | Li Zhang |
| Second Hospital affiliated to Zhejiang University | Zhejiang | Xiambao Liu |
| Shaoyifu Hospital affiliated to Zhejiang University | Zhejiang | Guosheng Fu |
| Union Hospital affiliated to Fujian Medical University | Fujian | Lianglong Chen |
| First Hospital affiliated to Xiamen University | Fujian | Weihua Ke |
| Quanzhou First Hospital | Fujian | Rong Lin |
| West China Hospital | Sichuan | Mao Chen, Yingqiang Guo |
| Sichuan Provincial Hospital | Sichuan | Jianhong Tao |
| Nanchong Central Hospital | Sichuan | Haoyu Wang |
| West-southern Medical University Hospital | Sichuan | Zhongcai Fan |
| Guizhou Provincial Hospital | Guizhou | Qiang Wu |
| First Hospital affiliated to Kunming Medical School | Yunnan | Tao Guo |
| Guangdong Provincial Hospital | Guangdong | Jianfang Luo |
| Sunyixian Hospital affiliated to Zhongshan University | Guangdong | Jingfeng Wang |
| Southern Hospital affiliated to Southern Medical University | Guangdong | Yuqing Hou |
| Shenzhen People Hospital | Guangdong | Shaohong Dong |
| Shenzhen Sunyixian Cardiovascular Hospital | Guangdong | Qiang Liu |
| Meizhou People Hospital | Guangdong | Wei Zhong |
| First Hospital affiliated to Guangxi Medical University | Guangxi | Weifeng Wu |
| A total of 69 hospitals from 26 provinces and 4 municipalities participated in the study. | | |

**Supplementary Table 2: Number of missing values and corresponding dispositions**

| Variables | No of missing values | Disposition |
| --- | --- | --- |
| Body Mass index | 2 (0.4%) | ﻿multiple imputation |
| Estimated glomerular filtration rate | 12 (2.6%) | ﻿multiple imputation |
| Left atrial size | 12 (2.6%) | ﻿multiple imputation |
| Left ventricular end-diastolic diameter | 10 (2.2%) | ﻿multiple imputation |
| Left ventricular ejection fraction | 3 (0.7%) | ﻿multiple imputation |

**Supplementary Table 3: Univariable analysis of the predictive factors of physicians’ decisions against intervention**

| Variables | Odds ratio and 95% confidence interval | P value | Enter the multivariate model for subsequent variable selection |
| --- | --- | --- | --- |
| Demographics |  |  |  |
| Age | 1.092 (1.050-1.137) | <0.001 | Yes |
| Male | 1.203 (0.656-2.208) | 0.551 | Yes |
| BMI | 1.021 (0.939-1.110) | 0.627 | No |
| Risk factors |  |  |  |
| Current Smoker | 0.776 (0.294-2.045) | 0.608 | No |
| Hypertension | 1.592 (0.872-2.907) | 0.130 | Yes |
| Diabetes | 2.847 (1.492-5.432) | 0.001 | Yes |
| Dyslipidemia | 1.198 (0.534-2.687) | 0.661 | No |
| Comorbidities |  |  |  |
| Coronary heart disease | 1.954 (1.036-3.684) | 0.038 | Yes |
| Myocardial infarction | 1.689 (0.359-7.943) | 0.507 | No |
| Previous PCI | 1.419 (0.471-4.273) | 0.534 | No |
| Previous CABG | 2.806 (0.286-27.508) | 0.376 | No |
| Atrial fibrillation | 1.742 (0.765-3.968) | 0.187 | Yes |
| Cardiomyopathy | NA | 0.990 | No |
| Aortic disease | 1.44 (0.662-3.132) | 0.358 | No |
| Cerebrovascular disease | 1.247 (0.464-3.353) | 0.662 | No |
| Peripheral artery disease | 1.419 (0.471-4.273) | 0.534 | No |
| COPD | 1.409 (0.400-4.968) | 0.593 | No |
| Renal insufficiency | 2.375 (1.239-4.553) | 0.009 | Yes |
| Malignant tumor | 2.589 (0.688-9.749) | 0.160 | Yes |
| Charlson comorbidity index | 1.341 (1.175-1.530) | <0.001 | Yes |
| Symptoms |  |  |  |
| Angina pectoris | 1.628 (0.875-3.030) | 0.124 | Yes |
| NYHA class |  |  | Yes |
| I | Reference | Reference |  |
| II | 1.182 (0.251-5.573) | 0.833 |  |
| III | 0.955 (0.207-4.410) | 0.953 |  |
| IV | 4.320 (0.926-20.147) | 0.063 |  |
| Syncope | 2.123 (0.959-4.701) | 0.063 | Yes |
| Investigations |  |  |  |
| LVEF | 0.950 (0.930-0.971) | <0.001 | Yes |
| LV | 1.024 (0.987-1.063) | 0.207 | Yes |
| LA | 1.055 (1.013-1.098) | 0.010 | Yes |
| Combined moderate AR | 0.558 (0.262-1.186) | 0.129 | Yes |
| Combined moderate MVHD | 1.722 (0.896-3.311) | 0.103 | Yes |
| Pulmonary hypertension | 1.960 (1.049-3.663) | 0.035 | Yes |
| EuroSCORE-II | 1.289 (1.201-1.384) | <0.001 | Yes |

**Abbreviations:** yr= year; no.= number; BMI= body mass index; PCI= percutaneous coronary intervention; CABG= coronary artery bypass grafting; COPD= chronic obstructive pulmonary disease; NYHA= New York Heart Association; LVEF =left ventricular ejection fraction; LV= left ventricular end-diastolic dimension; LA= left atrium end-diastolic dimension; AR= aortic regurgitation; MVHD= multiple valvular heart disease

**Supplementary Table 4: Univariable analysis of the predictive factors of 12-month mortality**

| Variables | Hazard ratio and 95% confidence interval | P value | Enter the multivariate model for subsequent variable selection |
| --- | --- | --- | --- |
| Therapeutic decisions |  |  | Yes |
| Decision to intervene | Reference | Reference |  |
| Patient refusal of intervention | 3.123 (1.438-6.786) | 0.004 |  |
| Physician denial of intervention | 7.730 (4.142-14.427) | <0.001 |  |
| Demographics |  |  |  |
| Age | 1.043 (1.005-1.082) | 0.025 | Yes |
| Male | 1.202 (0.677-2.136) | 0.530 | Yes |
| BMI | 0.932 (0.860-1.009) | 0.083 | Yes |
| Risk factors |  |  |  |
| Current Smoker | 0.937 (0.399-2.201) | 0.881 | No |
| Hypertension | 1.071 (0.612-1.875) | 0.811 | No |
| Diabetes | 2.155 (1.174-3.958) | 0.013 | Yes |
| Dyslipidemia | 1.898 (0.990-3.639) | 0.054 | Yes |
| Comorbidities |  |  |  |
| Coronary artery disease | 1.743 (0.959-3.167) | 0.068 | Yes |
| Myocardial infarction | 1.74 (0.423-7.164) | 0.443 | No |
| Previous PCI | 0.987 (0.307-3.174) | 0.982 | No |
| Previous CABG | NA | 0.995 | No |
| Atrial fibrillation | 3.549 (1.909-6.600) | <0.001 | Yes |
| Cardiomyopathy | 5.345 (0.737-38.772) | 0.097 | Yes |
| Aortic disease | 1.427 (0.693-2.942) | 0.335 | No |
| Cerebrovascular disease | 2.979 (1.487-5.969) | 0.002 | Yes |
| Peripheral artery disease | 0.972 (0.302-3.127) | 0.963 | No |
| COPD | 2.489 (0.987-6.279) | 0.053 | Yes |
| Renal insufficiency | 2.552 (1.416-4.597) | 0.002 | Yes |
| Malignant tumor | 0.687 (0.095-4.975) | 0.710 | No |
| Charlson comorbidity index | 1.279 (1.144-1.429) | <0.001 | Yes |
| Symptoms |  |  |  |
| Angina pectoris | 1.064 (0.573-1.978) | 0.844 | No |
| NYHA class |  |  | Yes |
| I | Reference | Reference |  |
| II | 0.638 (0.178-2.287) | 0.490 |  |
| III | 0.583 (0.169-2.015) | 0.394 |  |
| IV | 3.026 (0.899-10.185) | 0.074 |  |
| Syncope | 2.094 (1.016-4.317) | 0.045 | Yes |
| Investigations |  |  |  |
| LVEF | 0.954 (0.936-0.973) | <0.001 | Yes |
| LV | 1.047 (1.012-1.083) | 0.008 | Yes |
| LA | 1.087 (1.049-1.126) | <0.001 | Yes |
| Combined moderate AR | 0.658 (0.328-1.318) | 0.238 | Yes |
| Combined moderate MVHD | 2.251 (1.259-4.025) | 0.006 | Yes |
| Pulmonary hypertension | 1.717 (0.953-3.091) | 0.072 | Yes |
| EuroSCORE-II | 1.097 (1.055-1.142) | <0.001 | Yes |

**Abbreviations:**  yr= year; no.= number; BMI= body mass index; PCI= percutaneous coronary intervention; CABG= coronary artery bypass grafting; COPD= chronic obstructive pulmonary disease; NYHA= New York Heart Association; LVEF =left ventricular ejection fraction; LV= left ventricular end-diastolic dimension; LA= left atrium end-diastolic dimension; AR= aortic regurgitation; MVHD= multiple valvular heart disease

**Supplementary Table 5: Comparison of the multivariate models of physicians’ decisions against intervention generated using stepwise selection method and LASSO method**

|  | Stepwise selection model* | LASSO model† |
| --- | --- | --- |
| Discrimination |  |  |
| C-statistic | 0.887 | 0.884 |
| IDI (95%CI) | Reference | -0.0156 (-0.0459 to -0.0147)  P=0.312 |
| NRI (95%CI) | Reference | -0.1684 (-0.4633 to -0.1265)  P=0.263 |
| Calibration |  |  |
| LR test | Reference | P=0.035 |
| BIC | 257.4 | 255.7 |

**Abbreviations:** IDI= integrated discrimination improvement; NRI= net reclassification improvement; LR= Likelihood ratio; BIC= Bayesian information criteria.

*: Model generated using stepwise selection method on the basis of logistic regression model, including sex, left atrium end-diastolic dimension, and Euroscore-II

†: Model generated using LASSO-penalized logistic regression model, including left ventricular ejection fraction and Euroscore-II

**Supplementary Table 6: Comparison of the multivariate models of 12-month mortality generated using the stepwise selection method and the LASSO method**

|  | Stepwise selection model* | LASSO model† |
| --- | --- | --- |
| Discrimination |  |  |
| C-statistic | 0.803 | 0.792 |
| IDI (95%CI) | Reference | -0.006 (-0.064 to 0.037)  P=0.671 |
| NRI (95%CI) | Reference | -0.033 (-0.341 to 0.284)  P=0.871 |
| Calibration |  |  |
| LR test | Reference | P=0.036 |
| BIC | 553.3 | 551.0 |

**Abbreviations:** IDI= integrated discrimination improvement; NRI= net reclassification improvement; LR= Likelihood ratio; BIC= Bayesian information criteria.

*: Model generated using the stepwise selection method on the basis of Cox proportional hazard model, including therapeutic decisions, diabetes, atrial fibrillation, Cerebrovascular disease, renal insufficiency, and left ventricular ejection fraction.

†: Model generated using the LASSO-penalized Cox regression model, including therapeutic decisions, atrial fibrillation, left ventricular ejection fraction, Charlson comorbidity index, and Euroscore-II

**Supplementary Table 7: Standardized mean differences across covariates before and after IPW adjustment.**

|  | Standardized mean difference  before IPW adjustment | Standardized mean difference after IPW adjustment |
| --- | --- | --- |
| Intervention group vs. Patient-refusal group |  |  |
| Age | 0.385 | 0.001 |
| Sex | 0.111 | 0.103 |
| BMI | 0.202 | 0.053 |
| Coronary heart disease | 0.248 | 0.051 |
| Prior PCI | 0.302 | 0.031 |
| Prior CABG | 0.122 | 0.022 |
| Atrial fibrillation | 0.185 | 0.017 |
| Renal insufficiency | 0.068 | 0.028 |
| Charlson Comorbidity Index | 0.032 | 0.001 |
| NYHA III/IV | 0.088 | <0.001 |
| LVEF | 0.099 | 0.006 |
| EuroSCORE-II | 0.195 | 0.154 |
| Intervention group vs. Physician-denial group |  |  |
| Age | 0.559 | 0.188 |
| Sex | 0.104 | 0.052 |
| BMI | 0.045 | 0.027 |
| Coronary heart disease | 0.332 | 0.059 |
| Prior PCI | 0.136 | 0.021 |
| Prior CABG | 0.130 | 0.011 |
| Atrial fibrillation | 0.210 | 0.077 |
| Renal insufficiency | 0.376 | 0.129 |
| Charlson Comorbidity Index | 0.689 | 0.073 |
| NYHA III/IV | 0.173 | 0.120 |
| LVEF | 0.428 | 0.047 |
| EuroSCORE-II | 1.330 | 0.055 |

**Abbreviations:** BMI= body mass index; PCI= percutaneous coronary intervention; CABG= coronary artery bypass grafting; NYHA= New York Heart Association class; LVEF =left ventricular ejection fraction; IPW= inverse probability weighting

**Supplementary Table 8: Sensitivity analysis of patients whose actual treatments in line with their initial therapeutic decisions**

| **Study group** | **Therapeutic decisions** | **Univariate analysis of**  **12-month mortality** | | **Multivariate analysis of**  **12-month mortality using IPW†** | |
| --- | --- | --- | --- | --- | --- |
|  |  | **Crude HR (95% CI)** | **P value** | **Adjusted HR (95% CI)** | **P value** |
| **Whole cohort** | **Patient-refusal vs. Intervention** | 3.17 (1.46-6.89) | 0.004 | 2.61 (1.09-6.20) | 0.031 |
|  | **Physician-denial vs. Intervention** | 7.74 (4.15-14.45) | <0.001 | 7.30 (3.35-15.92) | <0.001 |
| **Sensitivity analysis group*** | **Patient-refusal vs. Intervention** | 4.69 (2.00-10.99) | <0.001 | 3.59 (1.35-9.52) | 0.010 |
|  | **Physician-denial vs. Intervention** | 11.37 (5.56-23.24) | <0.001 | 10.97 (4.65-25.88) | <0.001 |

**Abbreviations:** HR= Hazard ratio; CI= confidence interval

*: Patients whose actual treatments in line with their initial therapeutic decisions. In this setting, patients who initially refused the intervention proposal or were denied intervention by their physicians were censored at the time of intervention if they received intervention during the follow-up; patients who initially accepted the intervention proposal were excluded if they did not receive the scheduled procedures within the 1-year follow-up or died before operation.

†: ﻿Adjusted for age, sex, body mass index, coronary heart disease, prior percutaneous coronary intervention, prior coronary artery bypass grafting, atrial fibrillation, renal insufficiency, New York Heart Association class III/IV, left ventricular ejection fraction, Charlson Comorbidity Index, and EuroSCORE-II.

**Supplementary Figure**

**Supplementary Figure 1. Geographical distribution of hospitals participating in the China-DVD study**

**Supplementary Figure 2. Absolute standardized mean differences across covariates before (Green) and after (Red) IPW adjustment.** The plot illustrates the balance of covariates between the intervention group and the patient-refusal group (a.), and between the intervention group and the physician-denial group (b.) before and after IPW adjustment. Absolute standardized mean differences <20% (Dash line) indicates good comparability between the two groups.

**Supplementary Figure 3. ﻿Graphical inspection of scaled Schoenfeld residuals to test the proportional hazard assumption for 12-month mortality.** The graphs show the scaled Schoenfeld residuals against the transformed follow-up time. The solid line is a smoothing spline fit to the plot, with the dashed lines representing a 2-standard-error band around the fit.
